# Complete Genome Sequence of a GII.17 Norovirus Isolated from a Rhesus Monkey in China

**DOI:** 10.1128/genomeA.00904-16

**Published:** 2016-09-08

**Authors:** Bo Liu, Yufen Tao, Chao Li, Xintong Li, Jiansheng Liu, Zhanlong He, Ming Xia, Xi Jiang, Ming Tan, Hongqi Liu

**Affiliations:** a Institute of Medical Biology, Chinese Academy of Medical Science, Kunming, Yunnan, China; b Cincinnati Children’s Hospital Medical Center, Cincinnati, Ohio, USA; c Department of Pediatrics, University of Cincinnati College of Medicine, Cincinnati, Ohio, USA

## Abstract

The previously silent GII.17 norovirus was found to be the predominant genotype causing major epidemics in China in the 2014–2015 winter epidemic season. We report here the complete genomic sequence of a GII.17 norovirus (mky/GII.17/KM1509/CHN/2015) that infected rhesus monkeys at a monkey farm in southwestern China.

## GENOME ANNOUNCEMENT

Noroviruses (NoVs), members of the *Norovirus* genus within the family *Caliciviridae* (1), cause epidemic and sporadic acute gastroenteritis (AGE) in humans (2), leading to significant morbidity and mortality (3, 4). NoV contains a single-stranded, positive-sense RNA genome that is about 7.6 kb in length with a poly(A) tail at the 3′ terminus. The genome consists of three open reading frames (ORFs) encoding two structural and six or seven nonstructural proteins (5). The *Norovirus* genus is composed of six genogroups (GI to GVI), among which all GI, most GII, and a few GIV NoVs infect humans (6); but these human NoVs (huNoVs) are also detected in animals in some cases (7, 8). Based on the sequence of the major structural protein VP1, each genogroup of NoVs contains a few to 22 genotypes (9).

The NoVs that have been predominantly recognized as causing major epidemic AGE in humans worldwide for the past two decades are those belonging to genogroup II, genotype 4 (GII.4) (10). However, a previously silent GII.17 genotype was found to be the predominant strain causing major AGE epidemics in China and some Southeastern Asian countries during the 2014–2015 winter epidemic season (11–13). Increased epidemics caused by this newly emerged GII.17 variant have also been seen in other countries worldwide recently, including in the United States (14, 15).

We report here our detection and genome sequencing of a GII.17 NoV from the fecal sample of a rhesus monkey collected from the general population of a monkey farm in southwestern China, 2015 (Z. He, B. Liu, Y. Tao, C. Li, M. Xia, W. Zhong, X. Jiang, M. Tan, H. Liu, submitted for publication). This GII.17 NoV is named mky/GII.17/KM1509/CHN/2015 and is abbreviated as KM1509.

KM1509 was first detected from fecal samples of monkeys by employing the OneStep RT-PCR kit (Qiagen) using the primer pair P289/P290 that was designed to detect caliciviruses. A PCR fragment with 310 bp was obtained and confirmed to be a GII.17 NoV genome fragment by sequencing, followed by BLAST research. A new forward primer was designed based on the obtained sequence, and we were able to amplify a 3.1-kb fragment covering the end of ORF 1, the full lengths of ORF2 and ORF3, as well as the poly(A) tail of the NoV genome, when the oligo T primer was used. The remaining 5′ half of the genome was also obtained using the primers described in a previous study for amplification of the whole genomes of huNoVs (16). The complete sequence of the whole genome was *de novo* assembled via SerialCloner 2-6-1. The mky/GII.17/KM1509/CHN/2015 genome is 7,556 nucleotides in length and consists of three expected ORFs. Pairwise comparisons of nucleotide sequences between the monkey GII.17 NoV (KM1509) and the other 11 human GII.17 NoVs (KC597139, KT380915, KT253245, KR083017, KP998539, LC037415, AB983218, LC043168, KU561251, KU561256, and KT970377) indicated that the sequence distance is only 76.9% with indels between KM1509 and the GII.17 NoV isolated in 1978, while sequence distances from 97.2% to 99.2% were seen between KM1509 and the other 10 GII.17 NoVs. Further analysis of specific domains in this genome will be helpful for exploring the origin and evolution of KM1509.

### Accession number(s).

The nucleotide sequence for mky/GII.17/KM1509/CHN/2015 has been deposited in GenBank under the accession number KX356908.

## References

[1] Robilotti E, Deresinski S, Pinsky BA 2015 Norovirus. Clin Microbiol Rev 28:134–164. doi:10.1128/CMR.00075-14. 25567225 PMC4284304

[2] Glass RI, Parashar UD, Estes MK 2009 Norovirus gastroenteritis. N Engl J Med 361:1776–1785. doi:10.1056/NEJMra0804575. 19864676 PMC3880795

[3] Pringle K, Lopman B, Vega E, Vinje J, Parashar UD, Hall AJ 2015 Noroviruses: epidemiology, immunity and prospects for prevention. Future Microbiol 10:53–67. doi:10.2217/fmb.14.102. 25598337

[4] Haessler S, Granowitz EV 2013 Norovirus gastroenteritis in immunocompromised patients. N Engl J Med 368:971. doi:10.1056/NEJMc1301022. 23465122 PMC4793940

[5] Lambden PR, Caul EO, Ashley CR, Clarke IN 1993 Sequence and genome organization of a human small round-structured (Norwalk-like) virus. Science 259:516–519. doi:10.1126/science.8380940. 8380940

[6] Kroneman A, Vega E, Vennema H, Vinjé J, White PA, Hansman G, Green K, Martella V, Katayama K, Koopmans M 2013 Proposal for a unified norovirus nomenclature and genotyping. Arch Virol 158:2059–2068. doi:10.1007/s00705-013-1708-5. 23615870 PMC5570552

[7] Farkas T 2016 Natural norovirus infections in rhesus macaques. Emerg Infect Dis 22:1272–1274. doi:10.3201/eid2207.151740. 27314565 PMC4918157

[8] Tse H, Lau SK, Chan WM, Choi GK, Woo PC, Yuen KY 2012 Complete genome sequences of novel canine noroviruses in Hong Kong. J Virol 86:9531–9532. doi:10.1128/JVI.01312-12. 22879606 PMC3416109

[9] Zheng DP, Ando T, Fankhauser RL, Beard RS, Glass RI, Monroe SS 2006 Norovirus classification and proposed strain nomenclature. Virology 346:312–323. doi:10.1016/j.virol.2005.11.015. 16343580

[10] Siebenga JJ, Vennema H, Zheng DP, Vinjé J, Lee BE, Pang XL, Ho EC, Lim W, Choudekar A, Broor S, Halperin T, Rasool NB, Hewitt J, Greening GE, Jin M, Duan ZJ, Lucero Y, O’Ryan M, Hoehne M, Schreier E, Ratcliff RM, White PA, Iritani N, Reuter G, Koopmans M 2009 Norovirus illness is a global problem: emergence and spread of norovirus GII.4 variants, 2001–2007. J Infect Dis 200:802–812. doi:10.1086/605127. 19627248

[11] Fu J, Ai J, Jin M, Jiang C, Zhang J, Shi C, Lin Q, Yuan Z, Qi X, Bao C, Tang F, Zhu Y 2015 Emergence of a new GII.17 norovirus variant in patients with acute gastroenteritis in Jiangsu, China, September 2014 to March 2015. Euro Surveill 20:21157. doi:10.2807/1560-7917.ES2015.20.24.21157. 26111236

[12] Kim JS, Kim HS, Hyun J, Kim HS, Song W 2015 Molecular epidemiology of human norovirus in Korea in 2013. BioMed Res Int 2015:468304. doi:10.1155/2015/468304. 26421289 PMC4572438

[13] Matsushima Y, Ishikawa M, Shimizu T, Komane A, Kasuo S, Shinohara M, Nagasawa K, Kimura H, Ryo A, Okabe N, Haga K, Doan Y, Katayama K, Shimizu H 2015 Genetic analyses of GII.17 norovirus strains in diarrheal disease outbreaks from December 2014 to March 2015 in Japan reveal a novel polymerase sequence and amino acid substitutions in the capsid region. Euro Surveill 20:21173. doi:10.2807/1560-7917.ES2015.20.26.21173. 26159307

[14] Medici MC, Tummolo F, Calderaro A, Chironna M, Giammanco GM, De Grazia S, Arcangeletti MC, De Conto F, Chezzi C, Martella V 2015 Identification of the novel Kawasaki 2014 GII.17 human norovirus strain in Italy, 2015. Euro Surveill 20:30010. doi:10.2807/1560-7917.ES.2015.20.35.30010. 26530698

[15] Parra GI, Green KY 2015 Genome of emerging norovirus GII.17, United States, 2014. Emerg Infect Dis 21:1477–1479. doi:10.3201/eid2108.150652. 26196235 PMC4517714

[16] Xue L, Cai W, Wu Q, Zhang J, Guo W 2016 Direct sequencing and analysis of the genomes of newly emerging GII.17 norovirus strains in South China. J Appl Microbiol 120:1130–1135. doi:10.1111/jam.13052. 26756909

